# Cytoplasmic m^1^A reader YTHDF3 inhibits trophoblast invasion by downregulation of m^1^A-methylated IGF1R

**DOI:** 10.1038/s41421-020-0144-4

**Published:** 2020-03-10

**Authors:** Qingliang Zheng, Haili Gan, Fenglian Yang, Yongli Yao, Fan Hao, Ling Hong, Liping Jin

**Affiliations:** Clinical and Translational Research Center of Shanghai First Maternity and Infant Hospital, Tongji University School of Medicine, Shanghai, 201204 China

**Keywords:** RNA modification, Extracellular matrix

## Abstract

N^1^-methyladenosine (m^1^A) is one of the important post-transcriptional modifications in RNA and plays an important role in promoting translation or decay of m^1^A-methylated messenger RNA (mRNA), but the “reader” protein and the exact biological role of m^1^A remain to be determined. Here, we identified that nine potential m^1^A “reader” proteins including YTH domain family and heterogeneous nuclear ribonucleoprotein by mass spectrometry, and among them, YTH domain-containing protein 3 (YTHDF3), could bind directly to m^1^A-carrying RNA. YTHDF3 was then identified to negatively regulate invasion and migration of trophoblast. Mechanistically, we found that the m^1^A “reader” YTHDF3 bound to certain m^1^A-methylated transcripts, such as insulin-like growth factor 1 receptor (IGF1R), with the combination of iCLIP-seq (individual-nucleotide resolution ultraviolet crosslinking and immunoprecipitation high-throughput sequencing) and m^1^A-seq. Furthermore, YTHDF3 could promote IGF1R mRNA degradation and thus inhibit IGF1R protein expression along with its downstream matrix metallopeptidase 9 signaling pathway, consequently decreasing migration and invasion of trophoblast. Thus, we demonstrated that YTHDF3 as an m^1^A reader decreased invasion and migration of trophoblast by inhibiting IGF1R expression. Our study outlines a new m^1^A epigenetic way to regulate the trophoblast activity, which suggests a novel therapeutic target for trophoblast-associated pregnancy disorders.

## Introduction

Eukaryote RNAs are found to possess more than 100 different types of post-transcriptional modifications, which can regulate RNA splicing, localization, stability, binding, and translation^1–3^. Methylation at the N6 position of adenosine (m^6^A) is involved in the epigenetic regulation of gene expression^4,5^. N1-methyladenosine (m^1^A) is one frequently occurring modification, which is typically found at many transfer RNAs (tRNAs)^6,7^. Recent transcriptome-wide mapping also revealed that m^1^A modification is also present in human messenger RNAs (mRNAs) and suggested the potential roles of m^1^A in modulating mRNA splicing and translation; meanwhile, m^1^A modification is dynamically changed upon external heat or starve stimulation^8–10^. Maternal–fetal interface cells would suffer many external stimuli (hypoxia, lipopolysaccharide (LPS), and hormone), which dynamically changed during the pregnancy, especially the hypoxia environment could affect trophoblast activity^11–13^. However, whether the trophoblast has dynamic m^1^A modification upon hypoxia remains unclear. Furthermore, the exact function of dynamic m^1^A upon different stimuli, especially the m^1^A modification on specific nucleotide of unique mRNA, has still not been understood and remains to be explored in the future.

Previously, YTH domain-containing proteins were reported to bind directly to m^6^A and to regulate RNA metabolism as readers^14,15^. YTH domain-containing protein 3 (YTHDF3) and YTHDF1 promotes protein synthesis by interacting with the translation machinery and YTHDF3 can increase decay of m^6^A-bearing RNA^16,17^, but YTHDF2 decreased the stability of m^6^A-modified mRNA and inhibits the translation of mRNAs^18^. YTH domain containing 1 (YTHDC1) regulates mRNA splicing by modulating pre-mRNA splicing factors^19^. Taken together, the YTH domain-containing proteins regulate various biological processes via binding to m^6^A-modified RNA^20^. m^1^A as another important RNA modification has been found to be written by tRNA methyltransferase 6 (TRMT6)/TRMT61A complex (writers)^21,22^ and erased by a-ketoglutarate-dependent dioxygenase alkB homolog 3 (ALKBH3) or ALKBH1 (erasers) in human cells^9,23^. It has been suggested that YTH domain family can interact with m^1^A-carrying RNA, potentially functioning as an m^1^A reader^24^. However, cellular proteins acted as readers involved in binding to m^1^A-carrying mRNA remain to be explored, and how the key target transcripts of these m^1^A readers affect cell activity and which underlying pathways and mechanisms mediate these changes are still far from elucidated.

“Insulin-like growth factor (IGF) axis” plays a key role in regulating cell growth and survival and affecting virtually every organ in the body^25–27^. In the placenta, the IGF 1 receptor (IGF1R) is expressed in all cell types, including the trophoblast and villous endothelium^28^. Downregulation of placental IGF1R might be an important factor in pregnancies complicated by intrauterine growth restriction (IUGR)^29^. Recent report has also shown a significant downregulation of IGF1R protein levels in IUGR pregnancies compared to normal pregnancies^30,31^. Interestingly, IGF1R protein downregulation in IUGR placentas was accompanied by impaired activation of intracellular signaling molecules of the IGF1R^32,33^. Moreover, insulin/IGF1R signaling can induce the expression of membrane-type matrix metalloproteinase (MMP), which is the key regulator of cell invasion, migration, and tissue remodeling^34,35^. However, how to accurately regulate the expression of IGF1R, especially via the RNA epigenetic way, subsequently affecting the downstream signaling of IGF1R, still needs to be illustrated.

Herein, we found that YTHDF3 acted as an m^1^A “reader” protein, and YTHDF3 expression was significantly downregulated in HTR8/SVneo cells upon hypoxia treatment. YTHDF3 knockdown increased the expression of key molecule IGF1R via enhancing m^1^A-modified IGF1R mRNA stability, and consequently upregulated MMP9 expression, and finally promoted trophoblast migration and invasion in vitro. Together, our results may add insights to the m^1^A “reader” YTHDF3 as an important regulator of the IGF signaling pathway in promoting invasion of trophoblast, providing potential targets to control pregnancy-associated diseases induced by hypoxia.

## Results

### YTHDF3 selectively binds to m^1^A-containing RNA

**Fig. 1:**
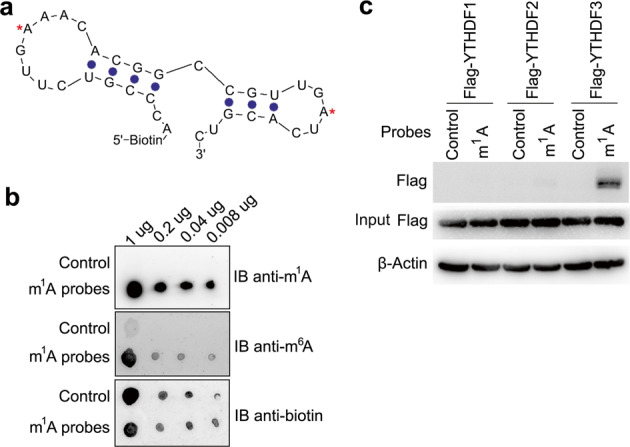
YTHDF3 selectively binds to m^1^A-carrying RNA. **a** Illustration of the m^1^A-modified RNA probe with stem and loop structure derived from the known m^1^A 1322 site in human 28S rRNA. Red asterisks indicated the m^1^A-modified sites. **b** Dot-blot analysis of the m^1^A probes specificity with m^1^A or m^6^A antibody. **c** IP and immunoblot analyses of the indicated probes in trophoblast HTR8/SVneo transfected with YTHDF1/2/3 plasmid as indicated. Data are representative of three independent experiments.

To better understand the biological functions of m^1^A in RNA, we designed a 5′-biotin-labeled m^1^A-carrying RNA sequence as the probe bait (Fig. 1a), which was derived from human 28S ribosomal RNA (rRNA) gene and harbored two m^1^A sites in the stem-loop structure^9^. First, the m^1^A and m^6^A contents of m^1^A probes were quantified by liquid chromatography with tandem mass spectrometry (LC-MS/MS), we found that the m^1^A/A ratio is about 30%, and m^6^A/A ratio is about 1% (Supplementary Fig. S1a), indicating that the m^1^A probes have no obvious m^6^A contamination. Furthermore, we verified that m^1^A antibody can specifically bind to m^1^A RNA probe, but not to the corresponding unmethylated control bait, and m^6^A antibody showed a faint nonspecific binding to m^1^A RNA probe (Fig. 1b). Using LC-MS/MS to screen systematically for cellular proteins that bound to m^1^A bearing RNA probe (Supplementary Fig. S1b, c), we identified 155 unique proteins in Raw264.7 cells and 27 unique proteins in HEK293T, respectively (Supplementary Fig. S1d, e). Among nine unique interaction proteins identified in both HEK293T and Raw264.7 cell lines (Supplementary Table S1), we found that YTHDF3 with the highest score in human cells can specificly bind to m^1^A modification probes (Supplementary Fig. S1f). Using biotin pulldown method, we confirmed that YTHDF3 bound significantly stronger to m^1^A RNA probes than YTHDF2, but YTHDF1 almost had no interaction (Fig. 1c); we further confirmed that YTHDF3 could bind to another m^1^A probe from SOX18 mRNA (Supplementary Fig. S1g). These results indicated that YTHDF3 selectively bound to m^1^A-methylated RNA and might act as a “reader” protein.

### YTHDF3 inhibited the activity of trophoblast

**Fig. 2:**
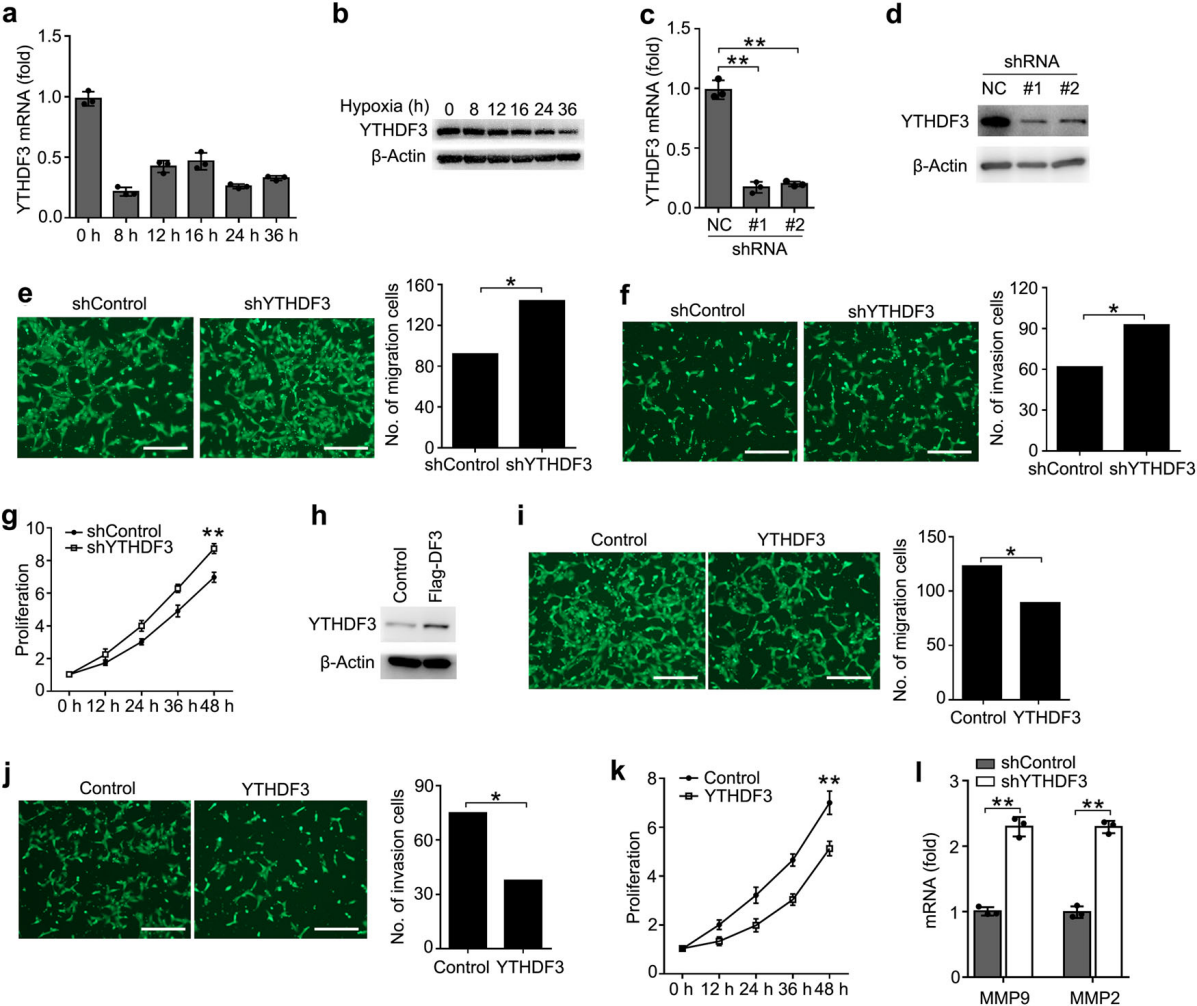
YTHDF3 inhibited the activity of trophoblast. **a**, **b** qRT-PCR and immunoblot analysis of YTHDF3 mRNA (**a**) and protein (**b**) in trophoblast treated with hypoxia as indicated time. The gene expression was normalized to that of the β-actin internal control in each sample. **c**, **d** qRT-PCR and immunoblot analysis of YTHDF3 in trophoblast HTR8/SVneo transfected with lentiviral shControl (NC), YTHDF3 shRNA #1 or #2 as indicated for 72 h. **e**, **f** Left: Migration (**e**) and invasion (**f**) analysis of HTR8/SVneo cells stably expressing control shRNA (shControl) vs. shRNA targeting YTHDF3 (shYTHDF3) by Transwell assay. Representative images are shown. Right: The number of migration and invasion cells was counted by the ImageJ software. **g**, **k** Cell proliferation measured by CCK8 assay of HTR8/SVneo cells stably expressing shYTHDF3 (**g**) and YTHDF3 (**k**) as indicated. The value of OD_450_ was normalized to the value about 24 h after cell seeding. **h** Immunoblot analysis of HTR8/SVneo cells stably expressing YTHDF3 as indicated. **i**, **j** Left: Migration (**i**) and invasion (**j**) analysis of HTR8/SVneo cells transfected with YTHDF3 plasmid by Transwell assay. Representative images are shown. Right: The number of migration and invasion cells was counted by the ImageJ software. **l** qRT-PCR analysis of MMP9 and MMP2 mRNA in HTR8/SVneo stably expressing shControl and shYTHDF3 as indicated. Scale bars, 100 μm; *p < 0.05, **p < 0.01 (Student’s *t* test). Data are representative of three independent experiments (mean and s.d. of technical triplicates (**a**, **c**, **g**, **k**, **l**)).

First, we found that YTHDF3 was significantly downregulated in HTR8/SVneo cells upon hypoxia treatment (Fig. 2a, b), indicating that YTHDF3 may play a potential role in regulating the activity of trophoblast. To investigate the biological significance of YTHDF3, we examined the effects of YTHDF3 knockdown on trophoblast activity. We confirmed that YTHDF3 was stably knocked down by using two different lentiviral short hairpin RNA (shRNA) sequences (Fig. 2c, d). We found that YTHDF3 knockdown significantly promoted the migration, invasion, and proliferation of trophoblast (Fig. 2e–g). In parallel, overexpression of YTHDF3 significantly inhibited the migration, invasion, and proliferation of trophoblast (Fig. 2h–k), suggesting that YTHDF3 could inhibit the activity of trophoblast.

To further determine the role of YTHDF3 in regulating the activity of trophoblast, we examined the downstream genes of YTHDF3 via transcriptome sequencing. The results indicated that 478 genes in HTR8/SVneo were significantly changed upon YTHDF3 stable knockdown (GEO Accession number: GSE135407) (Supplementary Fig. S2). Because extracellular matrix metallopeptidase played an important role in promoting trophoblast invasion^36^, we further confirmed that knockdown of YTHDF3 enhanced the expression of MMP9 and MMP2 expression in HTR8/SVneo (Fig. 2l). These results indicated that YTHDF3 inhibited the invasion of trophoblast probably via inhibiting the MMP9 and MMP2 expression.

### Identification of YTHDF3-binding RNAs by iCLIP-seq

**Fig. 3:**
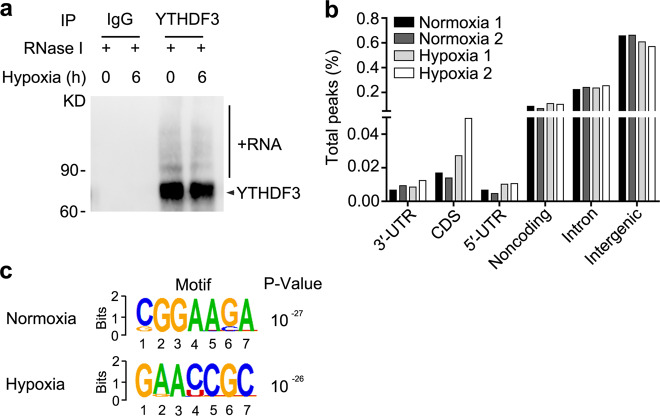
Identification of YTHDF3-binding RNAs by iCLIP-seq. **a** Immunoblot analysis of immuno-purified and biotin-labeled YTHDF3-RNA complexes transferred to PVDF membrane. IP with IgG as a control shows high specificity of the YTHDF3-RNA signal. **b** Percentage of total nucleotides under significant iCLIP-seq peaks within refSeq protein-coding genes broken down into transcript feature types extracted from refSeq. **c** Composite motif logo of the multiple sequence alignment of the most enriched hexamers under significant iCLIP-seq peaks within protein-coding genes as identified by *P* value, comparing hexamer frequencies to permutations of binding site locations within bound transcripts for normoxia (top) or hypoxia (bottom) cells. Data are representative of three independent experiments with similar results (**a**).

We further study the underlying mechanisms responsible for YTHDF3-mediated inhibition of trophoblast invasion. As YTHDF3 locates almost exclusively in the cytoplasm of HTR8/SVneo cells treated with hypoxia or LPS (Supplementary Fig. S3a) and it was reported previously that YTHDF3 functions primarily as the RNA-binding protein and m^6^A reader^17^, we hypothesized that cytoplasmic YTHDF3 might directly bind a series of RNAs to inhibit trophoblast invasion. To identify YTHDF3-binding RNAs or the direct targeted RNAs, we performed individual-nucleotide resolution ultraviolet (UV) crosslinking and immunoprecipitation (IP) high-throughput sequencing (iCLIP-seq). Immunoblotting of RNA-protein complexes revealed extensive signal for anti-YTHDF3 but not control anti-IgG IPs, suggesting the enrichment for YTHDF3-binding RNAs (Fig. 3a). Complementary DNA (cDNA libraries were constructed from the purified RNAs and submitted for Illumina deep-sequencing (Supplementary Fig. S3b, c). From four pooled YTHDF3 iCLIP experiments, we obtained 62 and 76 million clean reads from the HTR8/SVneo cells upon normoxia condition, and 72 and 40 million clean reads from hypoxia condition, respectively (GEO Accession number: GSE135642) (Supplementary Table S2). After mapping hg38 human genome and removing PCR duplicates, we yielded 889 and 1076 peaks from the normoxia cells, and 589 and 567 peaks from hypoxia cells (Supplementary Table S2). Interestingly, YTHDF3 peaks were mainly enriched in intergenic (~65%) and introns (~23%), but less (~12%) in coding sequence (CDS), 5′-untranslated regions (5′-UTRs), 3′-UTRs, and noncoding RNA regions (Fig. 3b), suggesting that YTHDF3 preferentially binds intergenic region of RNA transcripts. Most of the annotated RNA transcripts are mRNA and ribosomal RNAs, and 76 transcripts are shared between 131 ones in normoxia cells and 112 ones in hypoxia cells (Supplementary Fig. S3d, e, f), confirming that the data were highly consistent across different treatments. Hexamer enrichment analysis of iCLIP-seq peaks within all annotated genes identified that YTHDF3 bound to CGGAAGA motif of RNA transcripts in normoxia condition and GAACCGC motif in hypoxia condition (Fig. 3c). Together, these data showed both location and sequence preference for YTHDF3-binding RNAs.

To understand the functional targets of YTHDF3, we used DAVID (Database for Annotation, Visualization, and Integrated Discovery) to identify Kyoto Encyclopedia of Genes and Genomes (KEGG) and gene ontology (GO) terms enriched among protein-coding genes, which were associated with YTHDF3 iCLIP-seq peaks. We observed that YTHDF3 might take part in a set of biological processes and the enrichment for several cellular signaling pathways (Supplementary Fig. S3g, i). Intriguingly, YTHDF3-binding RNAs were enriched in insulin signaling pathway and vascular endothelial growth factor (VEGF) signaling pathway (Supplementary Fig. S3h, j). Thus, the iCLIP-seq peaks further reflect the signaling pathway of YTHDF3 in the regulation of trophoblast invasion.

### m^1^A-seq in trophoblast induced by hypoxia

**Fig. 4:**
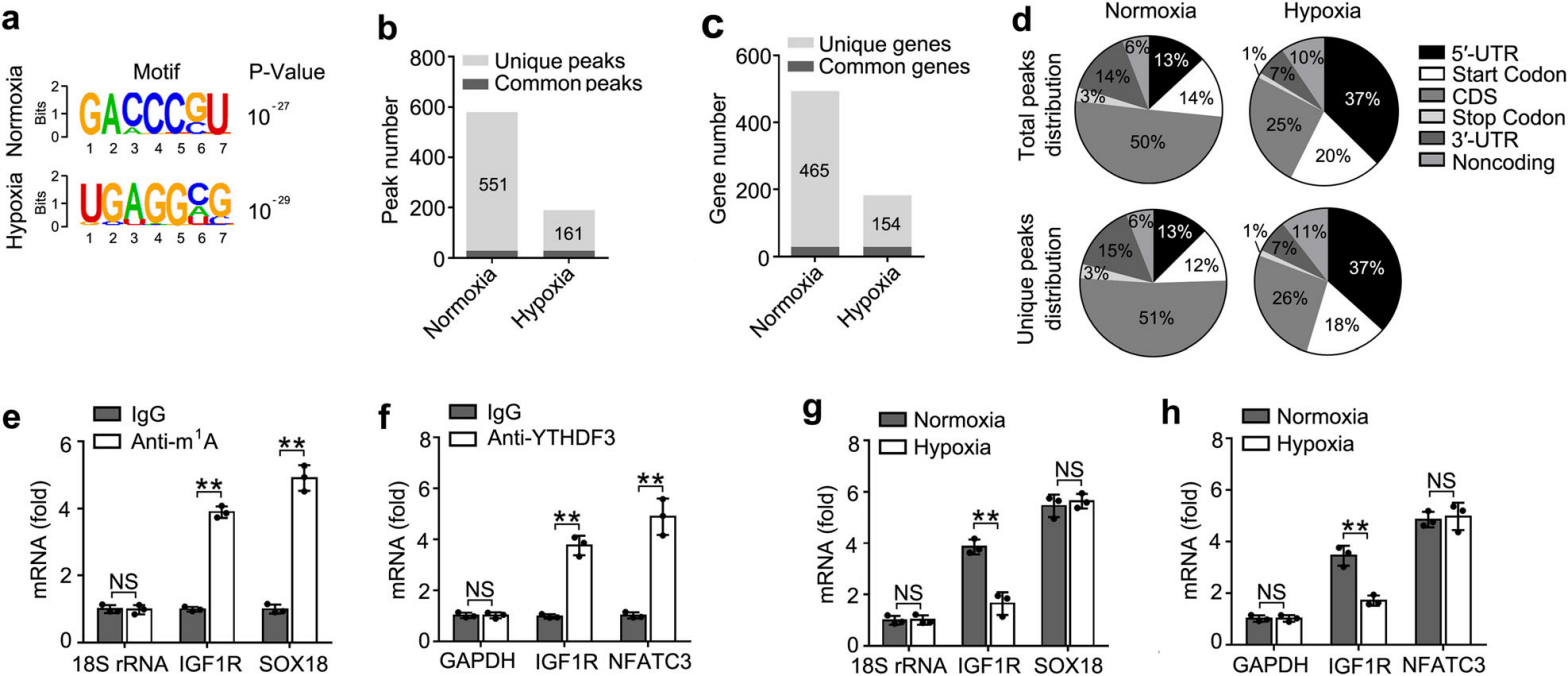
m^1^A-seq identified transcripts with altered methylation in trophoblast induced by hypoxia. **a** Composite motif logo of the multiple sequences with significant m^1^A peaks for normoxia (top) or hypoxia (bottom) HTR8/SVneo cells. **b** Number of m^1^A peaks identified in m^1^A-seq in normoxia and hypoxia HTR8/SVneo cells. **c** Number of m^1^A-modified genes identified in m^1^A-seq. Common m^1^A genes contain at least one common m^1^A peak, while unique m^1^A genes contain no common m^1^A peak. **d** Graphs of m^1^A peak distribution showing the proportion of total m^1^A peaks in the indicated regions in normoxia and hypoxia cells (top) and loss of existing m^1^A peaks (unique peaks in normoxia) or the appearance of new m^1^A peaks (unique peaks in hypoxia) after hypoxia treatment (bottom). **e**, **f** Enrichment fold of the indicated RNA transcripts in m^1^A (**e**), YTHDF3 (**f**) IP vs. RNA input control. **g**, **h** Enrichment fold of the indicated RNA transcripts in m^1^A (**g**), YTHDF3 (**h**) IP vs. RNA input control in HTR8/SVneo upon hypoxia for 12 h. Each transcript was quantified by qRT-PCR. NS: not significant; **p < 0.01 (Student’s *t* test); NS, not significant. Data are representative of three independent experiments (mean and s.d. of technical triplicates (**e**–**h**)).

To explore the mechanisms of m^1^A “reader” YTHDF3 on trophoblast activity, we intended to identify the transcripts with altered m^1^A methylation in trophoblast induced by hypoxia. We performed m^1^A-IP analysis of HTR8/SVneo cells upon hypoxia vs. normoxia (GEO Accession number: GSE135403) (Supplementary Table S3). Trophoblast upon hypoxia exhibited a relatively low total m^1^A level (Supplementary Fig. S4a). Consistent with previous m^1^A-seq results and that regions harboring m^1^A sites have higher GC content^8^, we identified that the m^1^A peaks were enriched near the start codons, with the relative conserved GACCCGU and UGAGGXG (X means C, A, U) motifs (Fig. 4a and Supplementary Fig. S4b). In total, we identified about 580 m^1^A peaks in about 494 transcripts from the normoxia sample and 190 m^1^A peaks in about 183 transcripts from the hypoxia sample, in which 29 transcripts showed common m^1^A modifications between the two groups (Fig. 4b, c). In HTR8/SVneo cells treated with hypoxia, 551 peaks in 465 transcripts disappeared and 161 new peaks in 154 transcripts appeared; the 29 peaks in 29 transcripts were found in both normoxia- and hypoxia-treated cells (Fig. 4b, c). We further investigated the m^1^A distribution patterns within both total and unique peaks. A similar pattern of total and unique m^1^A distribution in normoxia and hypoxia cells was observed when the RNA species were divided into 5′-UTR, start codon, CDS, stop codon, 3′-UTR regions of mRNAs, and noncoding RNAs (Fig. 4d). Interestingly, total or unique peaks in hypoxia showed a significant increase of m^1^A deposit appeared in 5′-UTR regions and a decrease of m^1^A in CDS regions compared to total peaks in normoxia (Fig. 4d). The transcripts showing disappeared (normoxia) or new (hypoxia) m^1^A methylation were enriched in GO terms related to cell migration and cell-matrix adhesion (Supplementary Fig. S4c, e), and KEGG terms related to RNA transport pathway, VEGF signaling pathway, and focal adhesion pathway (Supplementary Fig. S4d, f). These results of GO and KEGG analysis further indicated that m^1^A modification played important roles in the trophoblast cells mainly via regulating the cell-matrix signaling pathway.

Because the insulin signaling pathway played an important role in maintaining pregnancy^37^, we hypothesized that YTHDF3 might inhibit the activity of trophoblast through inhibition of the insulin pathway. Taken together with the sequencing results of transcriptome of YTHDF knockdown, iCLIP of YTHDF3 and m^1^A-IP, transcripts of IGF1R gene were among the most enriched YTHDF3-binding m^1^A-modified RNAs in insulin signaling pathway. Then, we performed IP of the HTR8/SVneo mRNA with m^1^A antibody, followed by quantitative reverse transcription PCR (RT-qPCR), and found that, in addition to negative control 18S rRNA, IGF1R mRNAs, and SOX18 (positive control)^8^ were enriched in the m^1^A antibody-immunoprecipitated fraction, and found that IGF1R were not enriched in the m^6^A antibody-immunoprecipitated fraction, confirming the presence of m^1^A, but no m^6^A sites on IGF1R transcripts (Fig. 4e and Supplementary Fig. S4g). Meanwhile, unlike glyceraldehyde 3-phosphate dehydrogenase (GAPDH), IGF1R and nuclear factor of activated T cells 3 (NFATC3) (positive control)^17^ were enriched in the YTHDF3 antibody-immunoprecipitated fraction, confirming that YTHDF3 can bind to IGF1R mRNAs (Fig. 4f). IGF1R mRNAs, but not SOX18 or NFATC3, showed decreased enrichment in the m^1^A or YTHDF3 antibody-immunoprecipitated fraction of the hypoxia cells compared to normoxia cells (Fig. 4g, h), thus indicating that m^1^A sites on IGF1R mRNAs disappeared upon hypoxia treatment and YTHDF3 failed to bind IGF1R mRNA. These results revealed that YTHDF3 regulated the activity of trophoblast probably via targeting IGF1R modification of IGF signaling pathway.

### YTHDF3 inhibited IGF1R expression

**Fig. 5:**
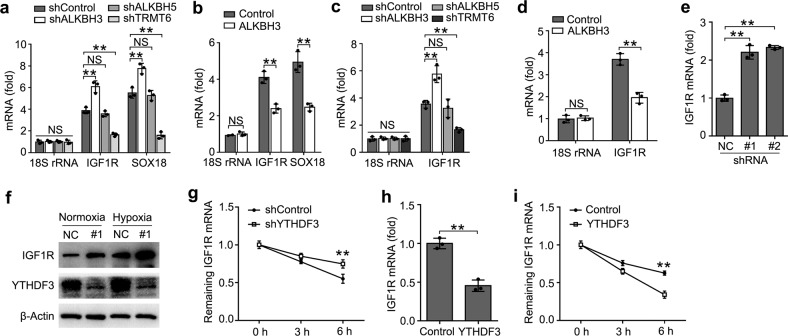
YTHDF3 inhibited IGF1R expression. **a**, **b** Enrichment fold of the indicated RNA transcripts in m^1^A IP vs. RNA input control in HTR8/SVneo after transfected with the indicated shRNA for 72 h (**a**) or plasmids (**b**) for 48 h. Each transcript was quantified by qRT-PCR. **c**, **d** Enrichment fold of the indicated RNA transcripts in YTHDF3 IP vs. RNA input control in HTR8/SVneo after transfected with the indicated shRNA for 72 h (**a**) or plasmids (**b**) for 48 h. **e**, **f** qRT-PCR and immunoblot analysis of the indicated mRNA and protein in HTR8/SVneo transfected with shYTHDF3 for 72 h. **g**, **i** RNA lifetime for IGF1R in HTR8/SVneo cells transfected with the shYTHDF3 (**g**) or YTHDF3 plasmids (**i**) was determined by monitoring transcript abundance after transcription inhibition (TI). **h** qRT-PCR analysis of the IGF1R in HTR8/SVneo transfected with plasmids as indicated for 48 h. NS: not significant; **p < 0.01 (Student’s *t* test); NS, not significant. Data are representative of three independent experiments (mean and s.d. of technical triplicates (**a**–**d**, **e**, **g**–**h**)).

As IGF1R mRNAs were methylated with m^1^A, we furtherly investigated the effects of IGF1R m^1^A modification after up- or downregulation of m^1^A-associated modification enzyme. We performed IP of the isolated trophoblast RNA with m^1^A antibody, and found that IGF1R mRNAs, especially positive control SOX18^8^, but not 18S rRNA showed increased enrichment in the m^1^A antibody-immunoprecipitated fraction when m^1^A eraser ALKBH3 was knocked down, decreased enrichment when the m^1^A writer TRMT6 was knocked down, but no effects on enrichment when m^6^A eraser ALKBH5 knocked down (Fig. 5a), thus indicating that m^1^A sites on YTHDF3-binding IGF1R mRNAs are the direct substrates subject to ALKBH3-catalyzed demethylation. Furthermore, the enrichment of IGF1R or SOX18 mRNAs to m^1^A antibody decreased in trophoblast after overexpression of ALKBH3 (Fig. 5b). These observations further support the notion that ALKBH3 can erase m^1^A modification of IGF1R mRNAs.

As YTHDF3 is an m^1^A reader of RNA methylation, we intended to investigate whether YTHDF3 directly targeted the m^1^A modification of IGF1R mRNA. We performed IP of the HTR8/SVneo RNA with YTHDF3 antibody, and found that IGF1R showed increased enrichment in the YTHDF3 antibody-immunoprecipitated fraction when ALKBH3 was knocked down, no change when ALKBH5 was knocked down, but decreased enrichment when the m^1^A “writer” TRMT6 was knocked down (Fig. 5c). Moreover, we found that IGF1R showed decreased enrichment in the YTHDF3 antibody-immunoprecipitated fraction after ALKBH3 overexpression (Fig. 5d). All these observations further supported that m^1^A sites on IGF1R mRNAs were the direct substrates of YTHDF3.

We next explored that whether the expression of IGF1R were affected after binding YTHDF3. We found that knockdown of *YTHDF3* increased the mRNA and protein levels of the *IGF1R* transcript (Fig. 5e, f). Accordingly, the IGF1R mRNA showed decreased RNA decay rates upon *YTHDF3* knockdown (Fig. 5g) and increased RNA decay rates upon ALKBH3 knockdown (Supplementary Fig. S5a), but there was no change on the mRNA and protein level of IGF1R upon knockdown of ALKBH5 (Supplementary Fig. S5b, c). Overexpression of YTHDF3 decreased the mRNA levels of the *IGF1R* transcript and the transcript showed increased RNA decay rates upon YTHDF3 overexpression (Fig. 5h, i). These results indicated that YTHDF3 inhibited the expression of m^1^A modified IGF1R via promoting the decay of the transcripts in the cytoplasm.

### YTHDF3 regulated the activity of trophoblast via targeting IGF1R

**Fig. 6:**
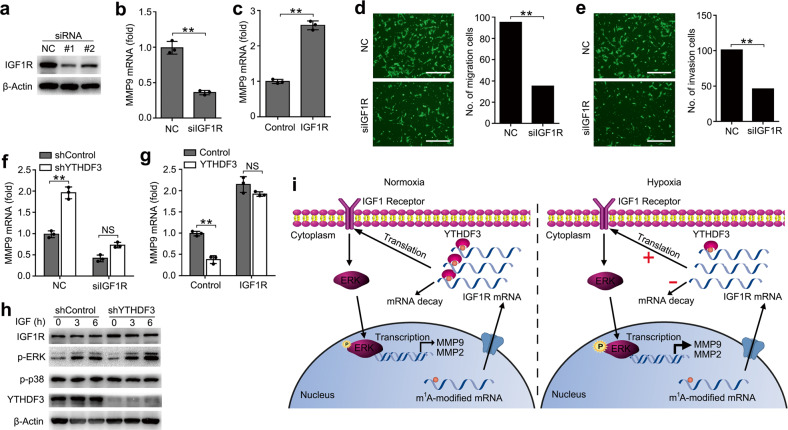
YTHDF3 regulated the activity of trophoblast via targeting IGF1R. **a** Immunoblot analysis of the indicated proteins in HTR8/SVneo transfected with IGF1R siRNA #1 or #2 as indicated for 48 h. **b**, **c** qRT-PCR analysis of MMP9 mRNA in HTR8/SVneo transfected with the indicated siRNA (**b**) or plasmid (**c**). **d**, **e** Left: Migration (**d**) and invasion (**e**) analysis of HTR8/SVneo cells transfected with NC vs. siIGF1R by Transwell assay. Representative images are shown. Right: The number of migration and invasion cells was counted by the ImageJ software. **f** qRT-PCR analysis of MMP9 mRNA in stably expressing shYTHDF3 HTR8/SVneo transfected with siIGF1R. **g** qRT-PCR analysis of MMP9 mRNA in stably expressing YTHDF3 HTR8/SVneo transfected with IGF1R overexpression plasmid. **h** Immunoblot analysis of the indicated proteins in HTR8/SVneo transfected with shYTHDF3, and stimulated with IGF cytokine for the indicated time. **i** Working model for the mechanism of YTHDF3 inhibiting trophoblast invasion by decreasing IGF1R expression via promoting m^1^A-carrying IGF1R decay. P: Phosphorylation. Scale bars, 100 μm; **p < 0.01 (Student’s *t* test); NS, not significant. Data are representative of three independent experiments (mean and s.d. of technical triplicates (**b**, **c**, **f**, **g**)).

We then investigated the potential roles of IGF1R in regulating the activity of trophoblast. First, we confirmed that IGF1R were efficiently knocked down by two different small interfering RNAs (siRNAs) (Fig. 6a and Supplementary Fig. S6a). We found that knockdown of IGF1R significantly decreased the production of MMP9 and MMP2 (Fig. 6b and Supplementary Fig. S6b), while overexpression of IGF1R could promote MMP9 and MMP2 expression (Fig. 6c and Supplementary Fig. S6c, d). Knockdown of IGF1R inhibited the invasion and migration of trophoblast (Fig. 6d, e) when compared with negative control, which was a phenocopy of our results obtained from overexpression of YTHDF3. Thus, IGF1R and YTHDF3 might act cooperatively in regulating the activity of trophoblast via IGF signaling pathway.

We next asked whether YTHDF3 regulated the activity of trophoblast mainly through IGF1R signaling. We found that YTHDF3 knockdown-mediated promotion of MMP9 production was rescued by IGF1R knockdown (Fig. 6f), suggesting that IGF1R knockdown counteracted the effect of YTHDF3 knockdown. Accordingly, overexpression of IGF1R reversed the YTHDF3 overexpression-mediated inhibition of MMP9 production (Fig. 6g), thus determining that the role of YTHDF3 in inhibiting trophoblast MMP9 production was dependent on IGF1R. We also found that the phosphorylation of extracellular signal-regulated kinase (p-ERK) increased in YTHDF3-knockdown cells and the total IGF1R protein expression also increased, in comparison with the control cells (Fig. 6h). Taken together, we found that YTHDF3 regulated the activity of trophoblast mainly via targeting IGF1R and affected downstream ERK-MMP9/2 signaling pathway.

On the basis of our findings, we propose the following working model to explain how m^1^A reader YTHDF3 inhibits the activity of trophoblast (Fig. 6i). Hypoxia treatment downregulates the m^1^A reader YTHDF3 expression and demethylates m^1^A modification of IGF1R, which both decrease the binding between YTHDF3 protein and IGF1R mRNA, and promote IGF1R expression in the cytoplasm, leading to the stronger activation of IGF1R signaling pathway, consequently enhancing MMP9 or MMP2 expression and promoting the invasion and migration of trophoblast cells.

## Discussion

We find that YTHDF3 can selectively bind to m^1^A-carrying RNAs, and hypoxia treatment can downregulate its expression in trophoblast HTR8/SVneo cells. Downregulation of YTHDF3 can enhance trophoblast migration and invasion in vitro. Mechanically, YTHDF3 inhibits the expression of IGF1R via binding m^1^A modification to enhance the decay of IGF1R mRNA, consequently inhibiting the expression of MMP9, and finally decreasing the invasion of trophoblast. Together, our results indicate that the m^1^A reader YTHDF3 may be a potential target to control pregnancy-associated diseases induced by hypoxia.

It is well known that YTH domain-containing proteins play as m^6^A readers and can regulate mRNA metabolism^15,38,39^, but whether they can act as readers of other RNA modifications is unknown. We use the m^1^A probes derived from the native present m^1^A modification site of human 28S rRNA with stem and loop structure^40^, because they can be more easily and specifically recognized by intracellular reader protein. We found that nine proteins, including mitochondrial ATP synthase subunit alpha (ATP5A1), 78 kDa glucose-regulated protein (HSPA5), transcriptional activator protein Pur-alpha (PURA), heterogeneous nuclear ribonucleoprotein M (HNRNPM), ADP/ATP translocase 2 (SLC25A5), RNA-binding protein 28 (RBM28), poly(rC)-binding protein 1 (PCBP1), YTH domain-containing family protein 3 (YTHDF3), and ruvB-like 2 (RUVBL2), can interact with m^1^A-carrying RNA. Inconsistent with the report that YTH domain-containing proteins (YTHDF1, YTHDF2, and YTHDF3) all probably play as m^1^A readers^24^, our data discriminate the differential binding affinities to m^1^A-carrying RNAs among them, suggesting different specificities probably endowed by unique protein structure of each one^41^. The functions of other eight “reader” proteins in regulating m^1^A-carrying mRNAs are completely unknown and warrant further investigation; nevertheless, our results provide a foundation for further understanding of the biological functions of m^1^A modification.

It is known that YTHDF3 can bind to a series of eukaryotic 40S and 60S subunits of RNAs and proteins, and then promote translation of m^6^A-carrying mRNAs^16,17^; we also found that most of the noncoding RNAs bound by YTHDF3 are ribosomal RNAs, including different 40S and 60S subunit RNAs, and speculate that YTHDF3 may also recruit 40S and 60S translation machineries to enhance the translation of m^1^A-carrying RNAs. We found that YTHDF3 peaks are mainly enriched in intergenic (~65%), introns (~23%), and noncoding RNAs (~10%), but merely (~2%) in CDS, 5′-UTR, and 3′-UTR regions, and our iCLIP results in trophoblast do not agree well with previous PAR-CLIP results in human HeLa or HEK293T cells^17,42^, probably due to our distinct cell line and different CLIP strategies.

Our m^1^A-seq results from normoxia and hypoxia along with our mechanistic studies in HTR8/SVneo cells reveal regulation of IGF1R expression as an important mediator of trophoblast invasion mediated by YTHDF3. Increased IGF1R expression is probably one of the main mediators of increased invasion in trophoblast with YTHDF3 downregulation, as overexpression of IGF1R is sufficient to rescue the inhibition of MMP9 expression mediated by YTHDF3 overexpression. These findings may be applicable beyond trophoblast invasion to other complicated pregnancy diseases or cancers driven by YTHDF3 overexpression. However, we cannot rule out the involvement of other signaling pathways that could be regulated directly or indirectly by YTHDF3.

We observed a global relative decrease in m^1^A mRNA methylation upon hypoxia, which could have effects on cellular physiology, especially if the methylation modifications of key transcripts are affected^43^. We found that many transcripts regarding insulin-like signaling pathway and extracellular matrix are demethylated upon hypoxia compared to normoxia, indicating that the reduced m^1^A methylation observed in trophoblast cells might affect functions associated with trophoblast invasion. We find that the key transcript IGF1R in IGF signaling pathway is demethylated upon hypoxia. A previous report indicated that m^1^A modification could enhance the translation of m^1^A-bearing mRNA, and YTHDF2, but not YTHDF1 or YTHDF3, could promote m^1^A-carrying mRNA decay and inhibit mRNA translation^23,24^. However, we found that YTHDF3 can promote m^1^A-bearing IGF1R decay and significantly inhibit IGF1R expression, and this effect is irrelevant to YTHDF3 functioning as an m^6^A reader. We speculate whether YTHDF3 targets on unique mRNA depends on its conserved binding motif and its m^1^A modification status. It was previously reported that YTHDF3 acted as an m^6^A reader^16^ and m^6^A modification plays an important role on physiological changes of trophoblast, and thus we think that YTHDF3 may also regulate the function of trophoblast via m^6^A modification, but its specific substrate RNA need to be further investigated. Furthermore, we found that m^1^A site on SOX18 is not affected in hypoxia condition or ALKBH5 overexpression. There might be other specific reader proteins for SOX18, which needs to be further investigated in the future.

Hypoxia has a critical role in the early embryo development and promotes the activity of trophoblast^44,45^. Abnormal oxygen condition can lead to injury or abortion of fetus (recurrent spontaneous abortion, IUGR)^46^. Expressions of various genes, especially ones involved in epigenetic modifications, are significantly regulated in hypoxia condition^47,48^. The hypoxia-inducible molecules have been proposed to be important regulators of first trimester trophoblast behavior^49^, and we found that YTHDF3 expression is downregulated in trophoblast upon hypoxia condition. These results are consistent with our data that downregulation of YTHDF3 promotes the invasion of trophoblast via upregulation of IGF1R expression, which finally increases MMP9 and MMP2 expression. Our results suggest that regulation of YTHDF3 expression could be a general control mechanism that affects a range of other biological processes, which will be a new direction to be explored in the future.

## Materials and methods

### Reagents

IGF1 (AFL291) was obtained from R&D System Company (Minneapolis, USA). Antibodies used for IP, immunoblot (IB) analysis and immunofluorescence (IF) were as follows: m^1^A antibody (D345-3, 1:100 for IP) was from MBL Life Science (Japan). Horse radish peroxidase-coupled secondary antibodies (ab6721, 1:1000 for IB), IGF1R (ab182048, 1:1000 for IB), ALKBH5 (ab69325, 1:1000 for IB), and YTHDF3 (ab83716, 1:1000 for IB) were from Abcam (Cambridge, UK). β-Actin (4970, 1:1000 for IB), p-ERK (4370, 1:1000), and p-p38 (4511, 1:1000) were from Cell Signaling Technology (Danvers, MA, USA). The Flag tags (F7425, 1:1000 for IB) as well as the agarose were used for IP; LPS (L2630) was from Sigma-Aldrich (St. Louis, MO, USA). DAPI (4′,6-diamidino-2-phenylindole) (62247, 1:1000 for IF) Alexa Fluor^®^ 546 (A-11071, 1:500 for IF) and Protein A/G Magnetic Beads (88802) were from Thermo Fisher Scientific.

### Cell culture and transfection

HTR8/SVneo cell lines were obtained from American Type Culture Collection (Manassas, VA, USA) and cultured as in RPMI-1640 supplemented with 10% fetal bovine serum (FBS) in normoxia (20% O_2_) or hypoxia (2% O_2_). HTR8/SVneo were transfected with siRNAs (final concentration: 20 nM) using INTERFERin (Polyplus-transfection) and plasmids with Lipofectamine™ 2000 (Thermo Fisher, American) according to the manufacturer’s instructions. All siRNAs were obtained from GenePharma (Supplementary Table S4).

To establish stably transfected HTR8/SVneo cells, G418 was added (1000 μg/mL) 48 h after transfection and maintained at 800 μg/mL for 3 weeks for positive selection. For stably transfected cells, YTHDF3 expressions were confirmed by Western blot. Lentiviruses harboring YTHDF3 shRNA or non-targeting control sequences were purchased from OBIO Technology (Shanghai, China). HTR8/SVneo cells were infected with the lentivirus at a virus titer of 10:1 to the cell numbers, and with 5 μg/mL polybrene (Sigma-Aldrich, USA) to enhance infection efficiency. After HTR8/SVneo cells were transfected for 72 h, puromycin was added (5 μg/mL) for 72 h and maintained at 2 μg/mL for 3 weeks for positive selection of stably knocked down YTHDF3. All cells were verified to be negative for mycoplasma contamination. The sequences encoding the shRNA are shown in Supplementary Table S5.

### RNA extraction and RT-qPCR

HTR8/SVneo cells were transfected with indicated shRNA, siRNAs, or plasmids. Total RNA was extracted by TRIZOL reagent (Invitrogen) 48 h post transfection. RNA was reversed transcribed using the Reverse Transcription System from TOYOBO (Osaka, Japan). The reverse transcription products from different samples were amplified by real-time PCR and analyzed as described previously^50^. The primer sequences for qPCR analysis are listed in Supplementary Table S6.

### RNA-seq and data analysis

The procedure was adapted from the previous report^51^. Total RNA was isolated using TRIZOL reagent (Invitrogen). cDNA library was constructed using TruSeq RNA Sample Prep Kit and then sequenced with HiSeq 2000 System (Illumina Inc.). The expression of transcripts was quantified as Reads Per Kilobase of exon model per Million mapped reads.

### Affinity purification of m^1^A-binding proteins and LC-MS/MS

Biotin-labeled oligo ribonucleotides with the sequence of 5′-biotin-ACCCGUCUUGXAACACGGCCGUUGXUCACGUC-3′ (X = m^1^A or A) from human 28S rRNA gene and 5′-biotin-CCGUUCCGCCCXGGCCGCGCCCAGCUGGAAUGCA-3′ (X = m^1^A or A) from SOX18 mRNA were obtained from Integrated DNA Technologies (IDT). HEK293T or Raw264.7 cells were lysed in cell lysis and binding buffer (containing 10 mM Tris-HCl (pH 7.5), 150 mM KCl, 1.5 mM MgCl_2_, 0.05% (v/v) NP-40, 10 mM NaCl, 0.5 mM DTT, 0.5% (v/v) Triton X-100, 2 mM EDTA, and 0.5 units RNase inhibitor) and centrifuged at 13,000 r.p.m. at 4 °C for 15 min. The supernatant was pre-cleared at 4 °C for 1 h by incubation with streptavidin-conjugated agarose beads (Thermo Scientific). The biotinylated m^1^A and control baits were incubated at 4 °C with pre-cleared cell lysates for 2 h. Streptavidin-conjugated agarose beads were then added to the mixture and incubated in a shaker at 4 °C for 2 h. The IP samples were separated on sodium dodecyl sulfate-polyacrylamide gel electrophoresis (SDS-PAGE) gels and stained with silver, different bands with intensive signal in m^1^A probes compared with control probes were cut and digested, and then analyzed by mass spectrometry LC-MS/MS experiments performed as previously described^50^.

### Transwell migration and invasion assay

For invasion assays, cell-culture inserts (0.8 μm, Falcon no. 353097) were pre-coated with Matrigel (40 μg/insert, Corning) in serum-free medium for 30 min at 37 °C. For migration assays, inserts were not pre-coated. Forty thousand cells per insert were seeded in the upper chamber of the insert and cultured in 300 μL RPMI-1640 medium supplemented with 2% FBS. Complete RPMI-1640 medium supplemented with 10% FBS was used in the lower chamber. Following 16–24 h of migration or invasion, fluorescent stain using calcein-AM was added to each lower chamber and incubated for 30 min. Images were collected with a Nikon Eclipse Ti2 with NIS Elements imaging software (version 5.02) and images were analyzed with ImageJ (version 1.51i).

### Cell proliferation assay

Five thousand cells were seeded per well in a 96-well plate. The cell proliferation was assessed by assaying the cells at various time points using the Cell Counting Kit-8 (Sigma-Aldrich, 96992) following the manufacturer’s protocols. For each cell line tested, the signal was normalized to the value observed about 24 h after seeding.

### m^1^A-seq and data analyses

Total RNA was isolated from normoxia or hypoxia HTR8/SVneo cell lines. Polyadenylated RNA was further enriched from total RNA using a Dynabeads mRNA Purification Kit (Invitrogen). RNA fragmentation, m^1^A-IP, and library preparation were carried out according to previously published protocols^9^. Sequencing was carried out on an Illumina HiSeq2500 machine in single-read mode with 50 base pairs per read. Significant peaks with false discovery rate <0.05 were annotated to the RefSeq database (hg38). Sequence motifs were identified using Homer. m^1^A-seq data were analyzed according to the protocol described previously^9^.

### m^1^A-qRT-PCR

The procedure was adapted from the previous report^8^. For m^1^A-qRT-PCR, total RNAs were firstly fragmented to 150 nucleotide using RNA fragmentation buffer (Ambion, AM8740), and then fragmented RNA (as input) was denatured and incubated with m^1^A antibody for 2 h, and protein A/G magnetic beads were added to the mixture and incubated for additional 2 h. Beads were washed with buffer and purified, input and immunoprecipitated RNAs were reversed transcribed into cDNA using RevertAid First Strand cDNA Synthesis Kit (Thermo Scientific), and then quantified by qPCR using SYBR GREEN mix (TOYOBO). For comparing m^1^A abundance changes, relative enrichment was first normalized with inputs, and then analyzed by comparing the data from m^1^A-immunoprecipitated sample. All samples were analyzed in triplicate qPCR.

### Measurement of RNA lifetime

HTR8/SVneo cells were seeded in 6-well plates at 50% confluency. After the cells were cultured for 24 h, actinomycin D (5 mg/mL, A4262, Sigma) was added for 6, 4, 2, and 0 h before collection. The total RNA was purified using Trizol method and RNA quantities were determined by qRT-PCR. The *GAPDH* gene was used as a reference gene when carrying out qPCR.

### Molecular cloning of related genes

Related genes were amplified from human trophoblast by RT-PCR and subsequently cloned into pcDNA vectors. Each construct was confirmed by sequencing. The corresponding primers used in this study are listed in Supplementary Table S7.

### Confocal microscopy

HTR8/SVneo cells plated on glass coverslips in six-well plates were treated in hypoxia condition for 12 h or stimulated with LPS for 6 h as indicated and fixed in 4% paraformaldehyde. Cells were then stained with YTHDF3 antibody and viewed using a Leica TCS SP2 confocal laser microscope.

### Immunoblot

Cells were lysed using Cell Lysis Buffer (Cell Signaling Technology) supplemented with cocktail protease inhibitor (Calbiochem). Protein concentrations of the extracts were measured using a BCA assay (Pierce, USA) and equalized with the extraction reagent. Equivalent amounts of extract were loaded to SDS-PAGE, transferred onto PVDF membranes, and then blotted as described previously^52^.

### Statistical analysis

All experiments were independently repeated at least three times. Comparisons between two groups were performed using Student’s *t* test. Data were analyzed with the GraphPad Prism 7 Software. Statistical values achieving *p* < 0.05 were considered to be statistically significant. There was no exclusion of data points. No randomization was used.

## Supplementary information

Supplementary information

## Data Availability

The source data that support the findings of this study are available from the corresponding author upon request.

## Supplementary information

**Supplementary Information** accompanies the paper at (10.1038/s41421-020-0144-4).
