# Supplementary material for: Cytoplasmic m1A reader YTHDF3 inhibits trophoblast invasion by downregulation of m1A-methylated IGF1R
Source: Cell Discov. 2020 Mar 10;6:12. doi: 10.1038/s41421-020-0144-4 (PMC7062805; doi:10.1038/s41421-020-0144-4)
Supplement: Supplementary file 1 — Supplementary information [file 41421_2020_144_MOESM1_ESM.pdf]

Supplementary information

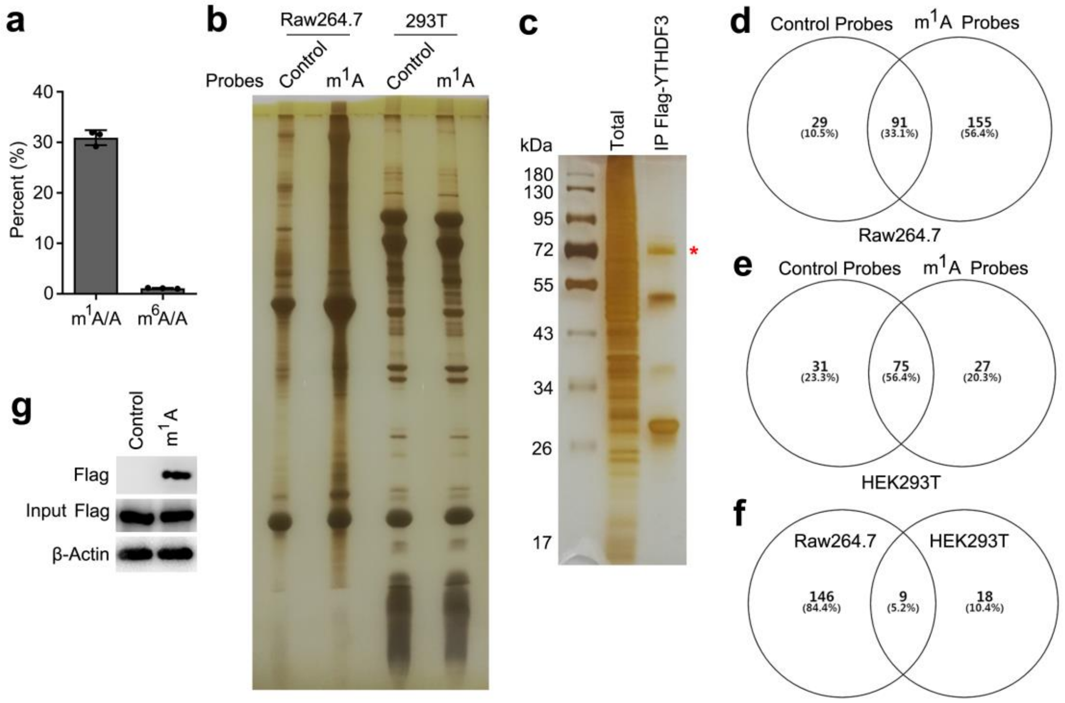

Supplementary Fig. 1 Identification of interacting proteins with m^{1}A modified probes. a The m^{1}A and m^{6}A contents of m^{1}A probes from human 28S rRNA gene were quantified by LC-MS/MS. **b** PAGE gel resolution of immunoprecipitated m^{1}A probes and its associated proteins from Raw264.7 and HEK293T by silver staining. Different bands were analyzed by MS. **c** PAGE gel resolution of immunoprecipitated YTHDF3 proteins from HEK293T by silver staining. Red * indicates the YTHDF3 bands. **d-f** Overlap of control probes and m^{1}A probes associated proteins in Raw264.7 (**d**), HEK293T (**e**), and overlap of common associated protein between Raw264.7 and HEK293T (**f**). **g** IP and immunoblot analyses of the indicated probes in trophoblast HTR8/SVneo transfected with YTHDF3 plasmid. Data are shown as representative photographs.

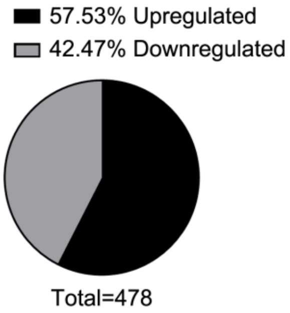

Supplementary Fig. 2 Transcriptome analysis of trophoblast upon YTHDF3 knockdown.

Graphs of the significantly changed genes in HTR-8/SVneo upon YTHDF3 knockdown.

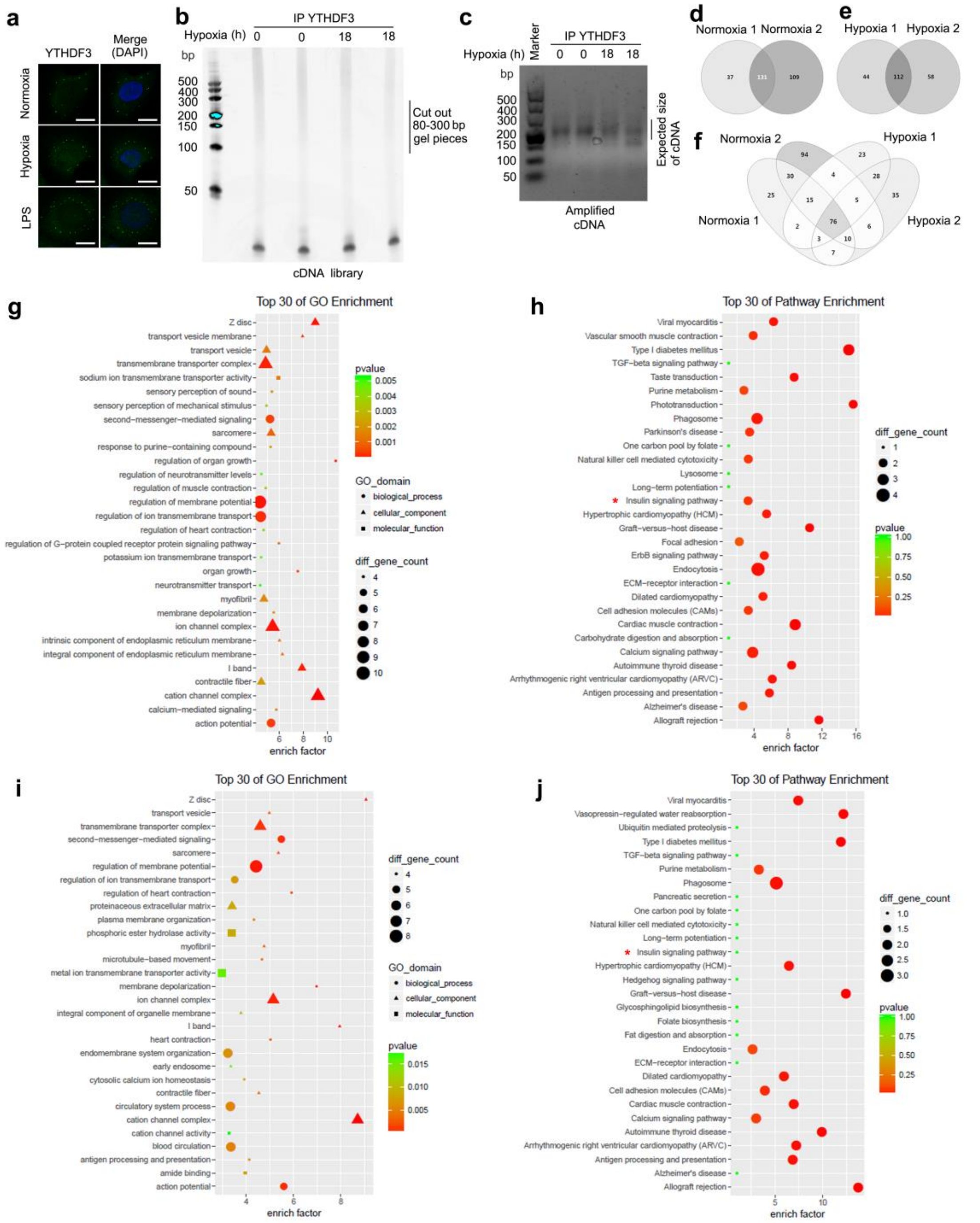

Supplementary Fig. 3 Gene Ontology (GO) and KEGG analysis of YTHDF3-binding mRNAs. a Confocal microscopy of HTR-8/SVneo treated in hypoxia for 12 h or stimulated with LPS (100ng/mL) for 6 h as indicated, then stained with YTHDF3 antibodies. Scale bar, 10 μm. **b** Gel electrophoresis analysis of cDNA treated as indicated for removing free RT primers and size selection. **c** Gel image of amplified cDNA libraries and extraction of the indicated size

ranges. **d,e** Overlap of two biological replicates for iCLIP in normoxia (**d**) or hypoxia (**e**) HTR-8/SVneo cells. **f** Overlap of transcripts identified by iCLIP of YTHDF3 in normoxia or hypoxia HTR-8/SVneo cells. Numbers in **d**,**e**,**f** show the sum of genes identified in each sample. **g**,**h** GO and KEGG analysis of the enriched YTHDF3-binding RNAs in normoxia cells but not in that of the hypoxia cells. **i,j** GO and KEGG analysis of the enriched YTHDF3-bound RNAs in hypoxia cell but not in that of normoxia cell. Red * indicates the enriched signaling pathway changed upon hypoxia. Data are representative of three independent experiments with similar results (**a**,**b**,**c**).

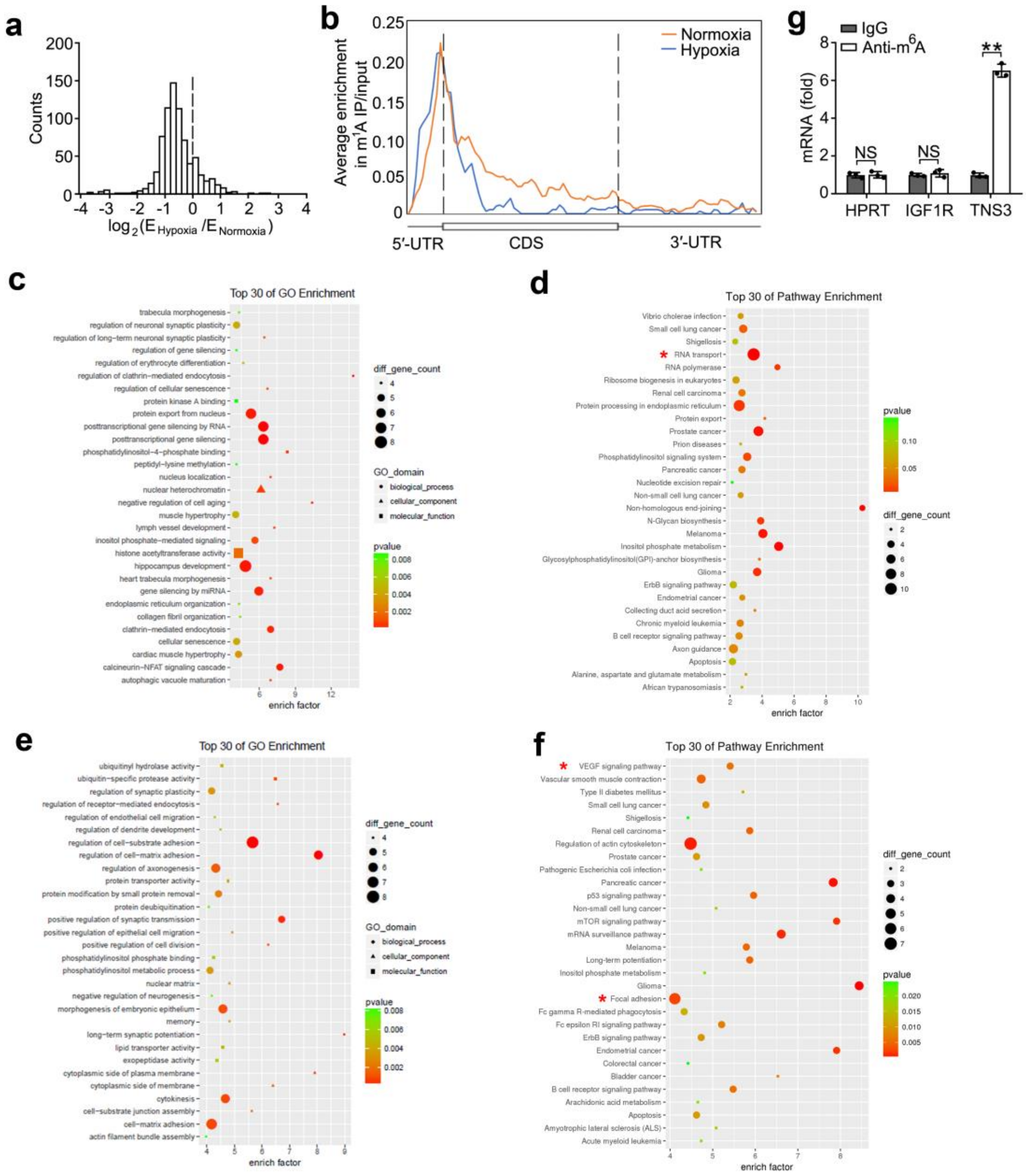

Supplementary Fig. 4 m¹A map of trophoblast induced by hypoxia identified by m¹A-seq. **a** Histograms showing the changes in m¹A enrichment in the hypoxia versus normoxia treated HTR-8/SVneo cells. **b** Metagene plots showing the average distribution of m¹A peaks identified across all transcripts in HTR8/SVneo treated with or without hypoxia for 12 h. **c,d** GO and KEGG analysis of the transcripts with disappeared m¹A modification in HTR-8/SVneo cells upon hypoxia. **e**,**f** GO and KEGG analysis of the transcripts with new occurred m¹A modification in HTR-8/SVneo cells upon hypoxia. **g** Enrichment fold of the indicated RNA transcripts in m⁶A IP versus RNA input control. Red * indicates the enriched signaling pathway upon hypoxia. NS: not significant; **p<0.01 (Student's t-test). Data are representative of three independent experiments (mean and s.d. of

technical triplicates (**g**)).

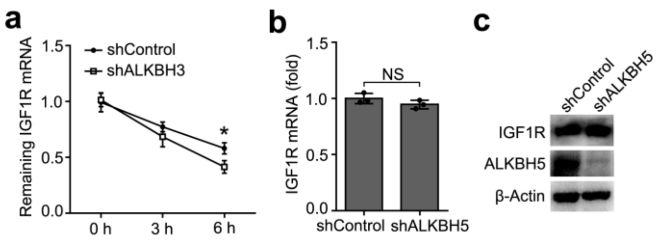

Supplementary Fig. 5 ALKBH5 knockdown did not affect the expression of IGF1R. a RNA lifetime for IGF1R in HTR-8/SVneo transfected with shControl or shALKBH3 was determined by monitoring transcript abundance after transcription inhibition (TI). **b** qRT-PCR and immunoblot analysis of IGF1R mRNA in HTR-8/SVneo transfected with shControl or shALKBH5 for 72 h. NS: not significant. *$p<0.05$ (Student's t-test). Data are representative of three independent experiments (mean and s.d. of technical triplicates (**a**,**b**)).

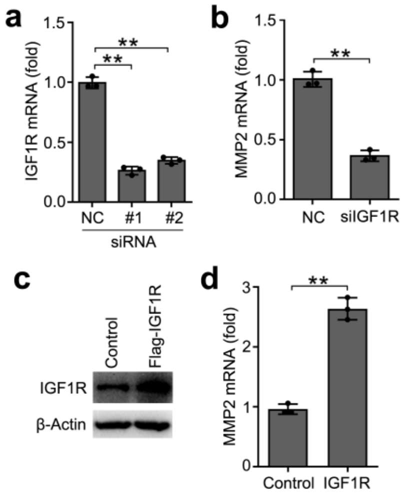

Supplementary Fig. 6 IGF1R mRNA was efficiently knocked down by RNA interfering. **a** qRT-PCR analysis of the IGF1R mRNA in HTR8/SVneo transfected with IGF1R siRNA #1 or #2 as indicated for 48 h. **b** qRT-PCR analysis of the MMP2 mRNA in HTR8/SVneo transfected with IGF1R siRNA #1 for 48 h. **c** Immunoblot analysis of the IGF1R protein in HTR8/SVneo transfected with IGF1R plasmid for 48 h. **d** qRT-PCR analysis of the MMP2 mRNA in HTR8/SVneo transfected with IGF1R plasmid for 48 h. **p<0.01 (Student's t-test). Data are representative of three independent experiments (mean and s.d. of technical triplicates (**a**, **b, d**)).

Supplementary Table S1 Overlap of proteins pulled down by m^{1}A probe between Raw264.7 and HEK293T.

Gene Name	Raw264.7			HEK293T		
	Score	Matches	emPAI	Score	Matches	emPAI
ATP synthase subunit alpha, mitochondrial (ATP5A1)	389	24(17)	0.8	41	4(2)	0.11
78 kDa glucose-regulated protein (HSPA5)	265	20(12)	0.56	32	4(2)	0.09
Transcriptional activator protein Pur-alpha (PURA)	132	7(6)	0.44	38	8(4)	0.44
Heterogeneous nuclear ribonucleoprotein M (HNRNPM)	116	8(4)	0.18	23	2(2)	0.09
ADP/ATP translocase 2 (SLC25A5)	90	14(4)	0.33	39	4(1)	0.1
RNA-binding protein 28 (RBM28)	65	6(2)	0.08	17	6(1)	0.04
Poly(rC)-binding protein 1 (PCBP1)	65	7(3)	0.18	152	6(5)	0.4
YTH domain-containing family protein 3 (YTHDF3)	35	5(3)	0.16	165	13(10)	0.49
RuvB-like 2 (RUVBL2)	34	3(2)	0.06	28	5(1)	0.06

Supplementary Table S2 Flowchart of YTHDF3 iCLIP-seq alignment and processing pipeline, resulting in peaks and genes.

	Normoxia		Hypoxia	
	Replicate 1	Replicate 2	Replicate 1	Replicate 2
Input	62,513,192	76,072,511	71,932,433	40,094,128
Mapped	47,366,424	56,910,397	55,228,446	28,010,060
Mapped/input	75.77%	74.81%	76.78%	69.86%
Peaks	889	1,076	589	567
Genes	168	240	156	170

Supplementary Table S3 Flowchart of m^1A-seq alignment and processing pipeline, resulting in peaks and genes.

	Normoxia		Hypoxia	
	Replicate 1 IP	Replicate 2 IP	Replicate 1 IP	Replicate 2 IP
Input	50,822,999	42,879,965	68,974,324	70,008,453
Mapped	48,913,621	41,257,415	66,451,984	67,095,701
Mapped/input	96.24%	96.22%	96.34%	95.84%
Peaks	580		190	
Genes	489		181	

Supplementary Table S4 siRNA sequences used for RNA interference

Human Genes		siRNA sequences (5′ to 3′)
IGF1R	sense #1	GCGGAGAGAUGUCAUGCAATT
	antisense #1	UUGCAUGACAUCUCUCCGCTT
	sense #2	GCUUCACCGUUUACUACAATT
	antisense #2	UUGUAGUAAACGGUGAAGCTT
Negative control	sense	UUCUCCGAACGUGUCACGUTT
	antisense	ACGUGACACGUUCGGAGAATT

Supplementary Table S5 Lentiviral shRNA sequences used for gene interference

Human Genes		shRNA sequences (5′ to 3′)
Ythdf3	target seq #1	CCTATGGACAAATGAGTAA
	target seq #2	GCAGTGGTATGACTAGCAT
Alkbh3	target seq	GGAACAGCTTTGTCAAGAT
Alkbh5	target seq	TCGTGTCCGTGTCCTTCTT
Trmt6	target seq	CTGAAACGTGAAGATGTGT
Negative control	target seq	TTCTCCGAACGTGTCACGT

Supplementary Table S6 Primers used for qRT-PCR

Human Genes		Primer sequences (5′ to 3′)
YTHDF3	Forward	TGACAACAAACCGGTTACCA
	Reverse	TGTTTCTATTTCTCTCCCTACGC
MMP2	Forward	CCCACTGCGGTTTTCTCGAAT
	Reverse	CAAAGGGGTATCCATCGCCAT
MMP9	Forward	TCCACCCTTGTGCTCTTCCC
	Reverse	CTGCCACCCGAGTGTAACCAT
IGF1R	Forward	AGTGCTGTATGCCTCTGTGAACC
	Reverse	ATAGACCATCCCAAACGACCC
18S rRNA	Forward	ACCCGTTGAACCCCATTCGTGA
	Reverse	GCCTCACTAAACCATCCAATCGG
HPRT	Forward	CCTGGCGTCGTGATTAGTGAT
	Reverse	AGACGTTCAGTCCTGTCCATAA
TNS3	Forward	CAGGGGTGGTAAAGGACGC
	Reverse	GGAGGGCTCCATTAAAGCTGAA
SOX18	Forward	AAAGCGTGGAAGGAGCTGAA
	Reverse	CGGTACTTGTAGTTGGGGTGGT
NFATC3	Forward	TATTTCGCACATCTTCATTACC
	Reverse	CCCTCGGCTACCTTCAGT
GAPDH	Forward	GCCAAGGTCATCCATGACAACTTTGG
	Reverse	GCCTGCTTCACCACCTTCTTGATGTC
Actb	Forward	CATGTACGTTGCTATCCAGGC
	Reverse	CTCCTTAATGTCACGCACGAT

Supplementary Table S7 PCR primers used for human genes cloning

Human Genes		Primer sequences (5' to 3')
YTHDF3	Forward	CCGCTCGAGATGTCAGCCACTAGCGTGGA
	Reverse	GGGAAGCTTTTATTGTTTGTTTCTATTTCTCTCC
YTHDF2	Forward	CCGCTCGAGATGTCGGCCAGCAGCCTCTT
	Reverse	GCCGGTACCTTATTTCCCACGACCTTGACGT
YTHDF1	Forward	CCGCTCGAGATGTCGGCCACCAGCGTGGA
	Reverse	GCCGGTACCTCATTGTTTGTTTCGACTCTGCC
IGF1R	Forward	CCGGAATTCATGAAGTCTGGCTCCGGAGGAGG
	Reverse	CGCGGATCCTCAGCAGGTCGAAGACTGGGGC
ALKBH3	Forward	ATAAGAATGCGGCCGCCAATGGAGGAAAAAAGAC GGCGAG
	Reverse	CACGGGAAGCTTTCACCAGGGTGCCCCTCGAG
